# Deficit-irrigation management for sustainable grape production (*Vitis vinifera* L.): different regimes to assess yield and berry quality under arid conditions

**DOI:** 10.1038/s41598-026-47407-8

**Published:** 2026-04-17

**Authors:** Abdel-Fattah M. El-Salhy, El-Nouby H. Salem, Moustafa M. A. Mohamed, Azza S. Hussein

**Affiliations:** 1 Pomology Department, Faculty of Agriculture, Assiut University, Assiut, Egypt; 2 Horticultural Department, Faculty of Agriculture and Natural Resources, Aswan University, Aswan, Egypt

**Keywords:** Flame seedless, Regulated deficit irrigation, Berry quality, Irrigation water productivity, Sustainable production, Ecology, Ecology, Environmental sciences, Plant sciences

## Abstract

Water scarcity is one of the main obstacles to agricultural development, particularly in Egypt. Grapevines are sensitive to drought and therefore depend heavily on improved irrigation regimes. It could be a crucial approach for the agricultural development of any country. This study evaluated the effect of deficit irrigation regimes on the growth, productivity, and cluster quality of the Flame seedless grapevines during two successive seasons (2024 and 2025) at a private orchard in the Luxor Governorate, Egypt. Grapevines were distributed in a completely randomized block design (CRBD) with three irrigation treatments and three replicates of each: (T1)—100% of crop water requirements (CWR), serving as the control; (T2)—80% of CWR; and (T3)—60% of CWR. The results indicated that vines receiving 80% CWR had a yield decrease of 3.70% compared to full irrigation; however, the amount of water saved throughout this process compensated for the lower yield, thus it did not have a significant overall impact. The irrigation treatment had an impact on all of the cluster’s assessed physicochemical characteristics, with irrigation at 80% CWR being the best strategy for reducing water consumption and enhancing cluster quality. By balancing resource conservation, productivity, and quality, 80% CWR represents a viable solution to meet the dual challenges of sustainable agriculture and water scarcity.

## Introduction

Grapevine (*Vitis vinifera* L.) is widely cultivated throughout the world and among the best fruit crops in terms of area and economic value^1^. Globally, grapevines cover approximately 7–7.5 million hectares, producing more than 77 million tons of grapes annually, with China, Italy, the United States, Spain, France, and Turkey being the largest producer countries^1^. Egypt is one of the top 15 nations in the world for grape exports. Grapes represent approximately 20.6% of Egypt’s total fruit exports, making them an important export fruit after citrus^2^. Flame Seedless is one of the most popular, widely cultivated, and economically important grape varieties in Egypt. It is particularly liked for its early ripening, high yield, and suitability for both local consumption and export markets^3^.

Water is a precious and scarce resource in Egypt. The availability of water for the agricultural sector is a significant barrier to agricultural productivity as the population expands. Installing a sufficient and effective irrigation system is one strategy to maximize this scarce resource. Orchards often use water-saving irrigation techniques, such as drip and microsprinkler systems, to optimize water resources while still producing high-quality fruit crops^4^.

In this regard, Egypt faces severe water shortages, making it one of the most water-stressed countries globally. Agriculture is the main consumer, using approximately 80–85% of Egypt’s available freshwater^5^. In general, in arid and semi-arid areas, which are most impacted by climate change, water availability is the most important and limiting factor for the expansion of agriculture and crop production^6^. Agriculture uses ~ 70% of global freshwater withdrawal, rising to ~ 90% in semi-arid and arid areas^7^. Many projections suggest that if demand management, reuse, and governance reforms are delayed, there will be large regional deficits and severe stress in key agricultural and urban areas by 2030–2050^8^.

Therefore, worldwide research attention has been directed towards identifying effective strategies to reduce water use during crop irrigation^9^. Deficit irrigation is a strategy in which a crop receives less water than it requires, and the resulting stress has minimal impact on crop yield. This effectively reduced water demand and improved plant water use efficiency (WUE)^10^ and fruit quality of various deciduous fruit trees, depending on the phenological stage when the water deficit was applied^11^.

Water scarcity is expected to increase with climate change, increasing the frequency and severity of drought events in major grape-producing regions^12^. Grapevines are widely known to be sensitive to water deficits, with drought causing reduced growth, reduced photosynthesis, cellular damage, and significant yield losses^13^.

Drought affects plant morphology, physiology, and biochemistry. Under such conditions, xylem vessels are exposed to embolism or dysfunction, leading to reduced hydraulic conductance and carbon uptake, which in turn affects plant growth characteristics and productivity^14^. When plants receive less water than they need, stomata close to reduce water loss, which limits CO₂ absorption and directly reduces photosynthetic activity. This results in a reduction in the production and export of sugars (carbohydrates), which are essential for plant growth, energy storage, and development processes^15^. Moderate water stress often reduces vegetative growth, causing a smaller canopy and greater light penetration to the buds. Increasing bud light exposure is closely associated with increased bud fruitfulness in grapevines and other fruit crops, since light promotes the development of inflorescence primordia and carbohydrate accumulation in buds^16^.

Moderate deficit can increase berry quality (sugar and phenols), but at the cost of yield^17^. Early-season deficiency affects berry size and yield, while late deficiency can increase berry composition^12^. Soluble solids (sugar content) often increase under moderate water stress due to berry dehydration and concentration effects^18^. Titratable acidity generally decreases as water loss increases, especially if stress occurs before veraison^19^. Drought stress, especially when applied before veraison (the beginning of ripening), results in consistently higher anthocyanin concentrations in grape skins across many varieties and environments. This increase is associated with upregulation of anthocyanin biosynthetic genes and is most pronounced under moderate water deficit. Water stress after veraison can also increase anthocyanin accumulation, especially some derivatives such as malvidin, but the effect is often less than stress before veraison^20^.

Generally, the management of available sources of water is of critical importance^21^. Actually, sandy, dry soils need a lot of irrigation because their high permeability reduces the quantity of water accessible to plants by causing deep seepage and quick infiltration below the root zone^22^.

Therefore, the specific objectives of the study were to (1) evaluate how deficit irrigation affects vegetative growth measurements, yield, and cluster quality attributes and (2) calculate grape irrigation water productivity (IWP) under various irrigation regimes. This contributes to the development of sustainable irrigation technologies that balance yield, quality, and resource conservation by providing useful recommendations for grape growers in arid regions.

## Materials and methods

This study was conducted on 19-year-old ‘Flame seedless’ grapevines (*Vitis vinifera* L.) grown in a private orchard located in Luxor Governorate, Egypt, for two successive seasons (2024 and 2025). Table 1 showed the experimental site’s climatic conditions that were arid with virtually absent rainfall^23^. At the beginning of the experiment, soil samples were taken at depths of 0–30 and 30–60 cm, and their physicochemical characteristics were examined (Table 2). The irrigation water was obtained from a deep groundwater well with an electrical conductivity (EC) of 1.5 dS m^− 1^ (Table 3). Every month, groundwater salinity levels were checked to ensure that they did not exceed the salinity threshold commonly used for irrigation water (EC less than 2250 µS cm^− 1^). To reduce evaporation and percolation losses, the irrigation system used a modern drip network with emitters (4 for each vine) spaced 0.5 m apart that supplied 4 L/h, and the operating time was set to deliver the calculated water (Table 4).

**Table 1:** Weather data of Luxor, Egypt during the 2024 and 2025 seasons.

Month	Maximum temperature (^°^C)		Minimum temperature (^°^C)		Humidity (%)		Cloud (%)		Wind speed (Kmph)		Rainfall (mm)	
	2024	2025	2024	2025	2024	2025	2024	2025	2024	2025	2024	2025
January	23	25	10	12	35	36	7	15	10.5	10.4	0	0
February	24	23	11	10	32	30	8	9	11.2	12.9	0	0
March	30	30	15	15	21	24	9	7	12.4	13.2	0.32	0
April	35	35	21	20	19	16	8	9	14.2	14.9	0.22	0
May	39	38	24	23	15	15	1	1	15.8	14.7	0	0
June	44	40	28	25	12	15	2	0	12.9	16.1	0	0
July	42	42	29	28	16	14	1	0	13.1	13.1	0	0
August	43	42	30	29	16	18	4	4	14.8	16.6	0	0
September	39	38	27	25	23	22	1	0	15.6	14.8	0	0
October	34	35	20	22	25	24	1	0	10.6	11.2	0	0
November	27	31	14	18	36	29	3	7	11.1	9.9	0	0

**Table 2:** Soil physicochemical analysis of the study area in the Luxor Governorate, Egypt.

Measured parameter	Unit	0–30 cm depth	30–60 cm depth
Electrical conductivity	dS m^− 1^	1.64	1.59
pH	-	8.44	8.38
Organic matter	%	0.083	0.067
CaCO_3_	%	16.87	15.75
Nitrogen (N)	%	0.03	0.02
Phosphorus (P)	ppm	4.77	4.16
Potassium (K)	ppm	152.06	178.32
Magnesium (Mg)	ppm	304.1	315.6
Calcium (Ca)	ppm	729.6	748.8
Copper (Cu)	ppm	1.05	0.97
Manganese (Mn)	ppm	1.91	1.76
Iron (Fe)	ppm	3.43	3.89
Sand	%	91.38	91.11
Silt	%	5.27	4.98
Clay	%	3.35	3.91
Texture	-	Sandy	Sandy
Field capacity	%	15.95	16.17

**Table 3:** Irrigation water analysis in the Luxor Governorate, Egypt.

Measured parameter	Unit	Value
Electrical conductivity	dS m^− 1^	1.5
pH	-	7.23
Sodium (Na^+^)	meq L^− 1^	1.0
Potassium (K^+^)	meq L^− 1^	2.1
Calcium (Ca^2+^)	meq L^− 1^	6.6
Magnesium (Mg^2+^)	meq L^− 1^	4.3
Chloride (Cl^−^)	meq L^− 1^	3.2
Sulfate (SO_4_^2−)^	meq L^− 1^	1.45
Bicarbonate (HCO_3_^−^)	meq L^− 1^	2.5

### Plant materials and irrigation treatments

Thirty-six vines, planted at 2 × 3 m in sandy soil, similar in growth vigor with no nutrient deficiency symptoms, were chosen for this experiment. The Spanish barron system was utilized as a trellising system. The vines underwent cane pruning, leaving 68 buds per vine, 10 canes with six buds each, and four renewal spurs with two buds. Apart from the irrigation, vines received the same agricultural practices as the entire orchard. They were distributed in a complete randomized block design (CRBD) with three irrigation treatments and three replicates, four vines of each: (T1)—100% of crop water requirements (CWR), serving as the control; (T2)—80% of CWR; and (T3)—60% of CWR.

Irrigation scheduling relied on crop evapotranspiration (ETc), which was calculated utilizing the Doorenbos and Pruitt^24^ equation:

$${\mathrm{ETc}} = {\mathrm{ET}}_{0} \times {\mathrm{Kc}}$$

where ET_0_ represents the reference evapotranspiration derived from weather data, and Kc is the local crop coefficient through the different phenological stages—vegetative growth: 0.2–0.4, flowering: 0.5–0.6, and fruit set until harvest: 0.7–0.9^25^. The local crop coefficient varies monthly depending on the plant’s phenological stages and the percentage of growth that the vine canopy shadows. Table 4 shows the total amount of water per vine during the season.

**Table 4:** Quantity of the irrigation water used during the 2024 and 2025 seasons.

Month	Irrigation frequency per month		Irrigation levels (L Vine^− 1^ Season^− 1^)		
			100% CWR (control)	80% CWR	60% CWR
January	4		120	96	72
February	8		264	211.2	158.4
March	12		360	288	216
April	12		432	345.6	259.2
May	12		468	374.4	281
June	16		576	460.8	345.6
July	16		576	460.8	345.6
August	16		576	460.8	345.6
September	12		360	288	216
October	12		360	288	216
November	8		264	211.2	158.4
December	4		120	96	72
Total water (m^3^ vine^− 1^ season^− 1^)			**4.48**	**3.58**	**2.69**
Total water (m^3^ ha^− 1^ season^− 1^)			**7466.5**	**5976.1**	**4483.2**

*CWR*: crop water requirements.

### Vegetative growth and leaf mineral content

Immediately after pruning (mid-January), the weight of the pruning wood was determined and expressed in kg/vine. Leaf samples were randomly collected by mid-June; the leaf area (cm²) for five mature leaves per vine was calculated according to Ahmed and Morsy^26^. Chlorophyll content (SPAD value) was estimated using the SPAD 502 Plus chlorophyll measuring device in the field for thirty-six leaves per treatment, and the average SPAD was calculated.

However, to determine the macronutrient content, leaf samples were collected, washed with distilled water, and then dried in an oven at 70^°^C. One gram of dried sample was pulverized using the mortar and pestle set, and the powder was digested with a solution of 350 ml H₂O₂, 0.42 g Se powder, 14 g LiSO₄H₂O, and 420 mL concentrated H₂SO₄^27^. Nitrogen (N), phosphorus (P), and potassium (K) were measured in each sample’s digest solution using the method outlined by Burt^28^.

### Yield and physical properties

At harvesting time (15ᵗʰ and 18ᵗʰ of May in the 2024 and 2025 seasons), the clusters of each vine were picked and weighed as a whole, and the yield/vine (kg) was recorded.

A random sample of four clusters was selected from the four directions of each vine to determine the physical properties. The weight of the cluster and berry (g) was determined using an electronic balance (Model ACS-A9, General, China).

The cluster length (cm) was measured using a measuring tape, and the number of berries in each cluster was determined. The compactness coefficient was then calculated based on Winkler et al.^29^.

### Berry chemical characteristics

Immediately after harvest, chemical analyses were conducted, and a sample of sixty berries was taken from the top, middle, and bottom of randomly chosen clusters from each vine. Berries were then homogenized in a blender; the juice obtained was filtered, and the total soluble solids (TSS%) were estimated at room temperature using a Galli 110 refractometer (Galli & C. S.R.L. Strumenti Scientifici, Merate, Italy). The titratable acidity percentage (g of tartaric acid per 100 mL of juice) was determined using the sodium hydroxide (0.1 N) titration method with phenolphthalein as an indicator^30^. The juice’s reducing sugar percentage was measured according to A.O.A.C^30^ protocol. Anthocyanins were extracted and measured by utilizing the spectrophotometer (Unico 1200-USA) at a wavelength of 535 nm, and values were expressed as mg/100 g fresh weight^31^.

### Irrigation water productivity (IWP)

The IWP is a key metric for assessing irrigation efficiency under each treatment, which was computed as the ratio of crop yield (kg/ha) to total water used (m³/ha)^32^.

### Statistical analysis

The study treatments were arranged in a complete randomized block design (CRBD), with three repetitions for each treatment and four vines per replication. The data were statistically analyzed using one-way analysis of variance (ANOVA) and the Least Significant Difference test at 0.05. A combined analysis was conducted over the seasons. The statistical software “Statistix v8.1,” CoHort Software (Pacific Grove, CA, USA), was used to compare the means of the irrigation treatments in accordance with Snedecor and Cochran^33^.

## Results

### Vegetative growth and leaf mineral content

The data in Table 5 stated the impact of regulated deficient irrigation levels on pruning wood weight, leaf area, and chlorophyll content during the 2024 and 2025 seasons. The results followed a similar trend during the two studied seasons. The findings reveal that increasing the irrigation water from 60 to 100% of crop water requirement (CWR) greatly improved the growth properties throughout the two years. There was no statistically significant difference in irrigation regulation between 80 and 100%. Furthermore, a decrease in the percentage weight of pruning wood (1.19% and 9.52%), leaf area (2.19% and 8.69%), and chlorophyll content (2.24% and 10.20%) was observed when using 80% or 60% CWR compared to 100% CWR, respectively.

**Table 5:** Effect of deficit irrigation water treatments on pruning wood weight (kg), leaf area (cm^2^), and chlorophyll content (SPAD) of Flame seedless grapevines in the 2024 and 2025 seasons.

Irrigation levels	Pruning wood weight (kg)			Leaf area (cm^2^)			Chlorophyll content (SPAD)		
	2024	2025	Mean	2024	2025	Mean	2024	2025	Mean
100% CWR	1.64a	1.71a	**1.68a**	147.6a	153.8a	**150.7a**	40.8a	39.6a	**40.2a**
80% CWR	1.62a	1.69a	**1.66a**	144.5a	150.2a	**147.4a**	40.1a	38.4a	**39.3a**
60% CWR	1.48b	1.56b	**1.52b**	134.9b	140.3b	**137.6b**	37.2b	35.1b	**36.2b**
LSD (*p* ≤ 0.05)	**0.09**	**0.12**		**8.15**	**9.11**		**1.88**	**1.91**	

*LSD*: Least significant difference utilized for mean comparisons; *CWR*: Crop water requirements.

Different letters in each column reveal significant differences at *p* ≤ 0.05.

Leaf macronutrient concentrations were generally related to the level of irrigation water, with the highest values recorded at 100% CWR (Table 6). However, concentrations at 100% and 80% CWR were insignificantly different during the two seasons, whereas the difference was significant compared to 60% CWR. Irrigation with 80 and 60% of CWR decreased the nitrogen by (2.42 & 13.94% N), phosphorus by (2.43 & 5.83% P), and potassium by (3.96 & 10.89% K), respectively, in comparison to 100%.

**Table 6:** Effect of deficit irrigation water treatments on leaf macronutrient contents (%) of Flame seedless grapevines in the 2024 and 2025 seasons.

Irrigation levels	*N*%			*P*%			K%		
	2024	2025	Mean	2024	2025	Mean	2024	2025	Mean
100% CWR	1.61a	1.68a	**1.65a**	0.201a	0.211a	**0.206a**	0.99a	1.02a	**1.01a**
80% CWR	1.57a	1.64a	**1.61a**	0.195a	0.206a	**0.201a**	0.95a	0.99a	**0.97a**
60% CWR	1.39b	1.45b	**1.42b**	0.189b	0.199b	**0.194b**	0.88b	0.92b	**0.90b**
LSD (*p* ≤ 0.05)	**0.06**	**0.08**		**0.009**	**0.010**		**0.06**	**0.08**	

*LSD*: Least significant difference utilized for mean comparisons; *CWR*: Crop water requirements.

Different letters in each column reveal significant differences at *p* ≤ 0.05.

### Yield and physical properties

The results in Table 7 illustrated that irrigation at 100% was the best at enhancing yield, but the application of 60% CWR resulted in the lowest yield during both seasons. Moreover, reduction in irrigation water usage from 100% to 80% did not result in any discernible changes. The yield/vine decreases were 3.70% and 17.11%, respectively, when 80% and 60% CWR were substituted for 100% CWR.

Moreover, all measured physical characteristics of grape clusters were affected by irrigation treatments in both seasons (Tables 7 and 8). Application of 100% or 80% CWR had a positive effect on all physical characteristics compared to the effects observed with 60% CWR. On the other hand, 60% CWR induced an increase of 15.08% and 11.35% of the cluster compactness coefficient in comparison to 100% and 80% CWR, respectively. It seems that improving the irrigation system by 80% of the total water utilized will improve the physical properties of the cluster. It also lowers production costs and the quantity of water used, which is very advantageous for water conservation. Additionally, it prevents soil salinity and a rise in ground water levels in the root zone.

**Table 7:** Effect of deficit irrigation water treatments on yield (kg/vine) and cluster weight (g), of Flame seedless grapevines in the 2024 and 2025 seasons.

Irrigation levels	Yield (kg/vine)			Cluster weight (g)		
	2024	2025	Mean	2024	2025	Mean
100% CWR	10.50a	11.12a	**10.81a**	350.1a	371.2a	**360.7a**
80% CWR	10.14a	10.67a	**10.41a**	338.1a	358.4a	**348.3a**
60% CWR	8.66b	9.25b	**8.96b**	301.8b	319.6b	**310.7b**
LSD (*p* ≤ 0.05)	**0.56**	**0.66**		**19.85**	**21.52**	

*LSD*: Least significant difference utilized for mean comparisons; *CWR*: Crop water requirements.

Different letters in each column reveal significant differences at *p* ≤ 0.05.

**Table 8:** Effect of deficit irrigation water treatments on cluster length (cm), compactness coefficient, and berry weight (g) of Flame seedless grapes in the 2024 and 2025 seasons.

Irrigation levels	Cluster Length (cm)			Compactness Coefficient			Berry Weight (g)		
	2024	2025	Mean	2024	2025	Mean	2024	2025	Mean
100% CWR	22.6a	21.9a	**22.3a**	5.76b	6.17b	**5.97b**	2.30a	2.53a	**2.42a**
80% CWR	21.9a	21.0a	**21.5a**	5.92b	6.41b	**6.17b**	2.13a	2.45a	**2.29a**
60% CWR	19.1b	18.3b	**18.7b**	6.59a	7.15a	**6.87a**	1.89b	2.05b	**1.97b**
LSD (*p* ≤ 0.05)	**1.18**	**1.25**		**0.38**	**0.46**		**0.21**	**0.26**	

*LSD*: Least significant difference utilized for mean comparisons; *CWR*: Crop water requirements.

Different letters in each column reveal significant differences at *p* ≤ 0.05.

### Berry chemical characteristics

**Fig. 1:**
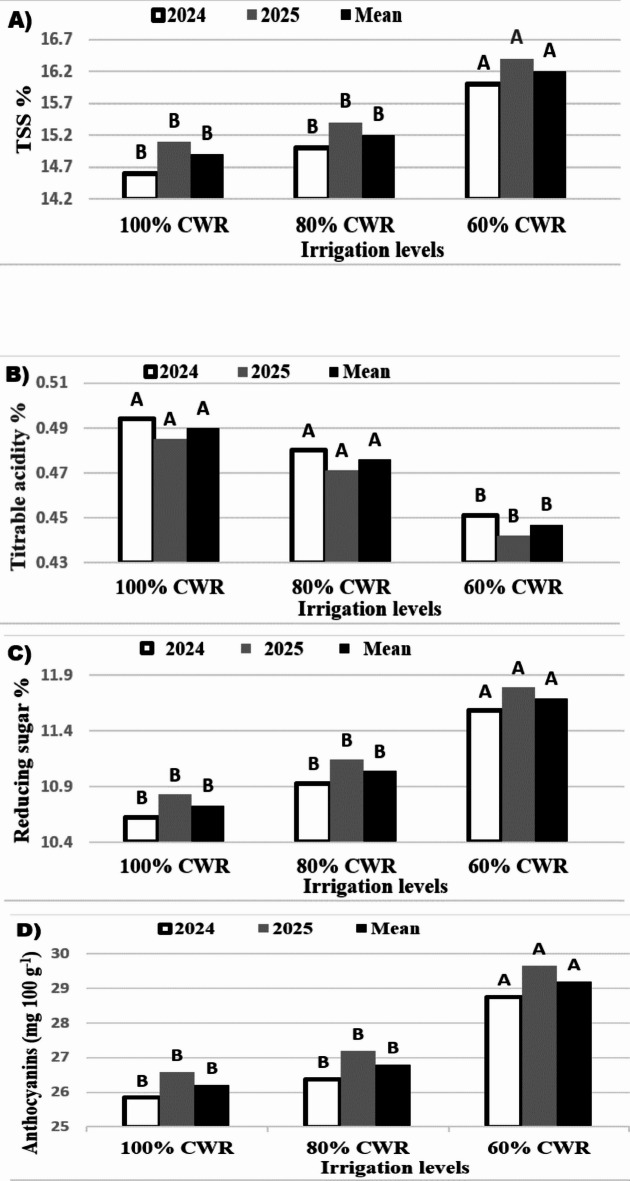
Effect of irrigation levels on the chemical quality characteristics (**A** total soluble solids, **B** titratable acidity, **C** reducing sugar, and **D** total anthocyanin) of the Flame seedless grape cultivar in the 2024 and 2025 seasons. Different letters reveal significant differences at *p* ≤ 0.05 (LSD test).

It is clear from the results in Fig. 1 that irrigation with 60% CWR significantly improved berry chemical characteristics in comparison to 80% or 100% CWR.

Based on Fig. 1, in both seasons, the soluble solids content in the berries increased with the reduction in CWR; utilizing 60% CWR had the best effect on titrable juice acidity when compared to the control group. Furthermore, berries from vines with 60% CWR had the highest levels of reducing sugar and total anthocyanin, followed by berries from vines with 80% CWR and berries from the control (100% CWR), which produced the lowest levels.

### Irrigation water productivity (IWP)

**Fig. 2:**
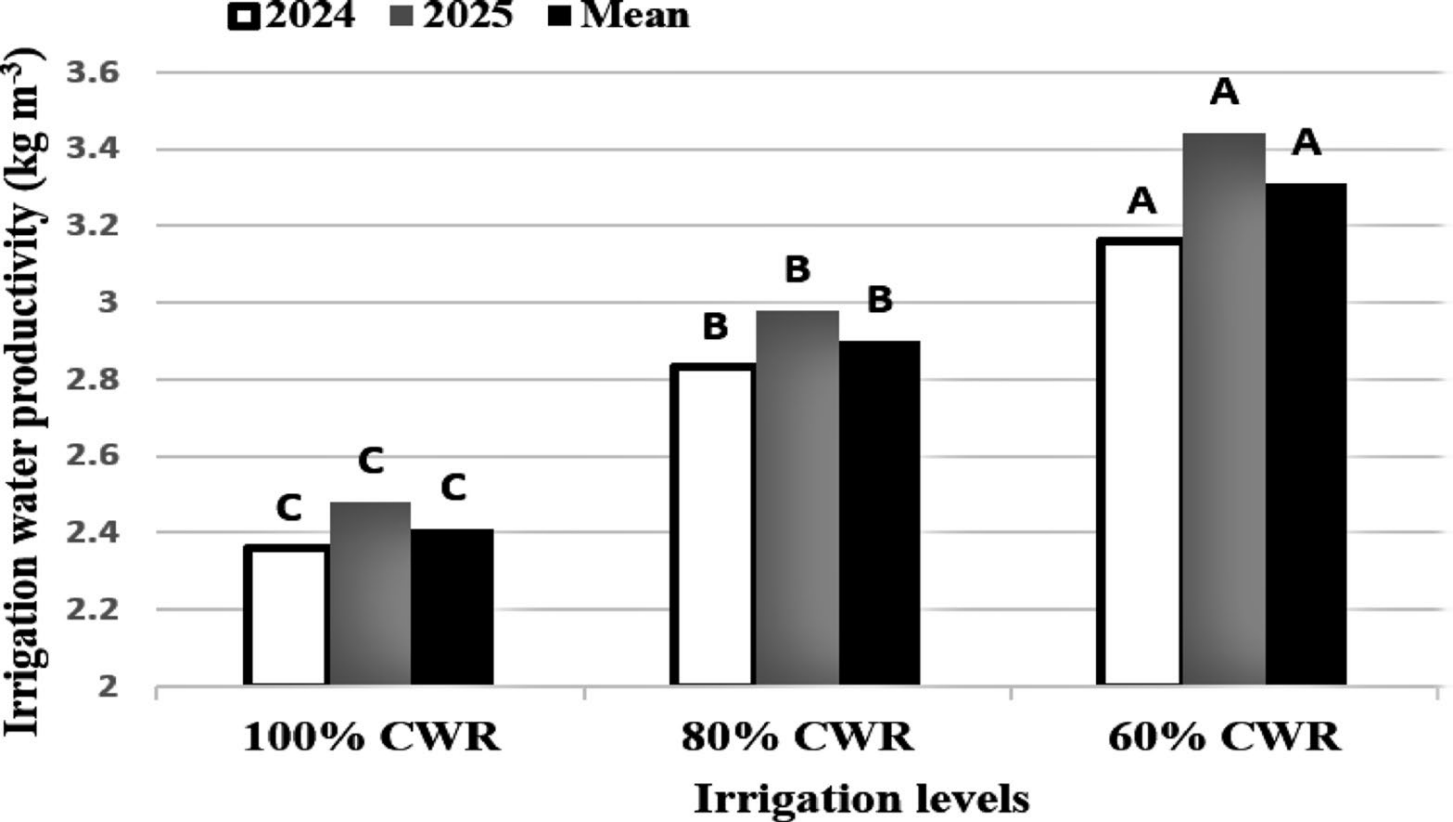
Irrigation water productivity for Flame seedless grapevines receiving different irrigation levels during the 2024 and 2025 seasons. Different letters reveal significant differences at *p* ≤ 0.05 (LSD test).

The irrigation water productivity (IWP) was the lowest at 100% CWR in comparison to deficit irrigation treatments during both years (Fig. 2). Grapevines received 7466.5 m^3^ ha^− 1^ season^− 1^ for the full irrigation system (100% CWR), 5976.1 m^3^ ha^− 1^ season^− 1^ for the 80% CWR, and 4483.2 m^3^ ha^− 1^ season^− 1^ for the 60% CWR. The IWP significantly increased to 20.33% for 80% CWR compared to 100% CWR and to 37.34% for 60% CWR compared to 100% CWR.

## Discussion

This study examines the potential effects of deficit irrigation regimes on the productivity and quality of the ‘Flame Seedless’ grape cultivar in arid conditions. One of the most important problems affecting agricultural productivity is drought, which is occurring more frequently worldwide. The availability of enough water for irrigation is one of the primary barriers to sustained fruit production in significant desert and semiarid growing regions^4,34^. Water scarcity is expected to increase with climate change, increasing the frequency and severity of drought events in major grape-producing regions^12^.

Vines that received full irrigation, 100% crop water requirements (CWR), had higher vegetative measurements in comparison to other vines (Table 5). This demonstrated that optimal water availability improved the growth of the vines and confirmed the role of water in expanding cells, enhancing the transportation of critical nutrients, and increasing photosynthetic activity, all of which are essential for optimal vegetative development^35^. Drip irrigation helps minimize nutrient loss as water passes through the lower layer of soil by maintaining the proper moisture level in the plant’s active root zone^36^. When compared to 100% CWR (control), vines receiving 80% CWR showed a slight decrease in vegetative growth, indicating that moderate water stress caused physiological adaptations that support continued growth. These adaptations probably include increased root-to-shoot ratios, enhanced water uptake efficiency, and decreased canopy transpiration, which allow trees under deficit irrigation to maintain growth while conserving water^37^. In grapevines under drought, petiole non-structural carbohydrates greatly increased compared to well-watered plants, and this was clearly interpreted as the participation of petiole sugars as osmo-compatible solutes in osmotic adjustment and turgor maintenance^38^. Regardless of sealed stomata, leaf CaOx crystals serve as inner carbon puddles that supply CO₂ for an initial stage of photosynthesis known as “alarm photosynthesis.” It prevents photoinhibition and the oxidative danger caused by carbon starvation under deleterious conditions^39^. Furthermore, severe drought greatly reduces stomatal conductance and photosynthetic rates, disrupts carbon supply, and often depletes starch reserves used for metabolism, osmotic adjustment, and hydraulic repair^40^. Hormonal imbalance associated with water stress leads to a decrease in both cell proliferation and leaf expansion^41^. A considerable reduction in transpiration rates, as well as impaired active transport and membrane permeability, which reduce the roots’ capacity to absorb nutrients, account for reduced leaf element levels at lower irrigation levels.

Moderate water stress often reduces vegetative growth, causing a smaller canopy and greater light penetration to the buds^12^. Increasing bud light exposure is closely associated with increased bud fruitfulness in grapevines and other fruit crops, since light promotes the development of inflorescence primordia and carbohydrate accumulation in buds^16^. On the other hand, both the number and size of inflorescences decrease when water is limited. Reduced photosynthesis can cause carbohydrate shortages, potentially hindering energy availability for inflorescence differentiation^42^. Severe water stress diminishes the ability to produce crops, inhibits evaporation, reduces shoot length, lowers yields, and degrades fruit quality^43^.

The different methods of irrigation had an effect on yield (Table 7). All the vines that received 100% CWR achieved maximum productivity, paving the way for providing sufficient, or rather abundant, quantities of water for fertile growth. The water case in grapevines affects the development and differentiation of inflorescence primordia through direct and indirect effects on biosynthetic processes and biochemicals, particularly in preserving cell turgidity, boosting photosynthetic activity, and facilitating the movement of nutrients and photo assimilates. Grapevines are resistant to moderate water stress, as evidenced by the fact that vines receiving 80% CWR produced almost 96% of the yield of vines under 100% CWR. Because there was less competition for resources, vines treated with moderate deficit irrigation probably allocated more resources to reproductive organs, maintaining yields even when water intake was decreased^44^.

The yield per vine increased when growth improved under 80% or 100% of the CWR quantity. Our results align with those of Intrigliolo and Castel^45^, who revealed that the yield generally enhanced in proportion to the total amount of water applied, primarily due to the larger berries. Irrigation application also boosted vine vegetative growth, with pruning wood weights showing a linear response to total water.

 El-Halaby^25^ found that when irrigation levels were 80%, 100%, or 120% of the estimated amount of water (EAW) rather than 60%, pruning wood weight, leaf area, nutritional status, and yield of Flame Seedless and Superior Seedless grapevines dramatically increased. There was no discernible increase in these growth traits when EAW was raised from 80 to 120%.

Furthermore, Permanhani et al.^46^ reported that deficit irrigation can stabilize the yield per vine while conserving water and controlling the vine. In addition, irrigation increased vine vigor and productivity, as evidenced by greater pruning wood weight and leaf area per vine, particularly in the more irrigated treatments^47^.

According to El-Salhy et al.^48^, pomegranate shoot length, leaf number, and leaf area were significantly affected by the irrigation schedules. Additionally, they found that the maximum yield was achieved by the highest moisture availability.

Deficiency irrigation treatments affected the berry chemical composition (Fig. 1). Vines receiving 80% CWR or 60% CWR produced berries with higher quality attributes compared to 100% CWR, with 60% CWR vines having the highest values ​​of TSS, reducing sugars, and anthocyanins. Water stress can accelerate the conversion of starch to sugar and cause sugars to accumulate in fruit due to diminished dilution effects^11^. Long-term moderate stress in Cabernet Sauvignon increased berry glucose and fructose, while berry weight and malic acid were reduced^49^. Titratable acidity generally decreases as water loss increases, especially if stress occurs before veraison^19^. Water stress raises secondary metabolism, particularly the synthesis of anthocyanins, as part of the stress response^50^. This increase is associated with upregulation of anthocyanin biosynthetic genes and is most pronounced under moderate water deficit^20^.

The irrigation water productivity showed a distinct upward trend from 100% CWR to 60% CWR, indicating improved IWP with decreased irrigation quantities (Fig. 2). Deficit irrigation strategies generally improve irrigation water productivity and can reduce plant water use by adjusting total leaf area while maintaining or enhancing fruit quality^51^. This was consistent with^52,53^, who reported that while 75% FC provided the greatest balance for water saving and production maintenance, lowering irrigation to 60% FC reduced yield. The primary physiological mechanisms governing the precise regulation of stomatal closure and photosynthesis under water stress and recovery encompass plant and leaf hydraulics as well as mesophyll conductance to CO₂^54^. Water deficit produces hydraulic and chemical signals, such as reduced root water potential, increased xylem sap pH, and/or increased abscisic acid (ABA) concentrations, all of which are involved in the control of stomatal opening and thus affect water use^55^. When the water supply to vine canopies is reduced, transpiration decreases more than it does in field crops, resulting in more net water savings^56^. Under mild water stress, grapevine photosynthesis is depressed almost entirely by stomatal closure, thereby increasing water use efficiency (the ratio of photosynthesis to transpiration)^57^. Moreover, this increase in irrigation water productivity indicates the adequacy of the recommended irrigation regime (80% CWR) for grape cultivation under arid conditions. Our results also showed that irrigation water productivity is a good indicator for determining the degree of drought tolerance in grapes under deficit irrigation. Through various processes, including maintaining physiological function, improving cluster quality, and increasing irrigation water productivity, the grapevines irrigated with 80% of CWR showed moderate tolerance to water deficiency.

## Conclusion

In this study, we investigated the effects of different irrigation regimes on the quality of the Flame seedless grape cultivar. Reducing the water supply to 80% CWR enhances vegetative growth and cluster quality when compared to clusters produced by 60% and 100% CWR applications. By balancing resource conservation, productivity, and quality, 80% CWR represents a viable solution to meet the dual challenges of sustainable agriculture and water scarcity.

## Data Availability

The datasets used and/or analysed during the current study available from the corresponding author on reasonable request.
